# Efficient federated graph aggregation for privacy-preserving GNN-based session recommendation

**DOI:** 10.1038/s41598-025-08256-z

**Published:** 2025-07-02

**Authors:** Jing Lou, Cheng Rong, Hanshen Chen, Daxue Liu

**Affiliations:** College of Intelligent Transportation, Zhejiang Institute of Communications, Hangzhou, China

**Keywords:** Computer science, Information technology, Software

## Abstract

Graph Neural Networks (GNN) have attracted increasing attention due to their efficient performance in recommendation systems. However, applying GNNs in session-based recommendations with emerging federated learning (FL) for a privacy-preserving recommendation is challenging. Firstly, constructing a global graph in a centralized manner is forbidden due to the privacy-preserving constraints of FL. Secondly, local graphs in each device contain minimal information on the global graph, causing the inefficient merging of sub-graphs by aggregating local models. Thirdly, the session data in these separated devices are usually extraordinarily non-Independent and Identically Distributed (non-IID), which harms the model performance. In this paper, we bridge the practical gaps between FL and GNN-based session recommendations for the first time by introducing a novel adaptive federated learning method named Federated Graph Aggregation (FedGA). FedGA is beyond the reach of prior adaptive FL methods by incorporating Divergence Resistant Aggregation (DRA) and Conditional Second-Moment Estimation (C-SME), yielding an efficient aggregator where local models trained by the unseen local graph embedding can be efficiently merged. Thanks to the above-proposed strategies, FedGA optimizes models without being interfered with by the aggressive learning rates generated by existing adaptive methods under extreme non-IIDness. In addition, we perform the theoretical analysis of the proposed method, and the results demonstrate that our method achieves a similar rate of convergence as other adaptive FL methods. We validate our method on both open datasets and real-world production data. Results show that our method obtains state-of-the-art performance compared to existing adaptive FL methods while retaining the comparable performance of the centralized methods.

## Introduction

In recent years, session-based recommendation (SR) methods have attracted increasing attention^1–22^. These methods predict the next item that a user most probably clicks on or buys without requiring the user/item features. However, different aspects, for example, time, environment, and social relationships, can affect user interests, causing a significant shift in user preferences. Graph Neural Network (GNN) has recently attracted attention for achieving state-of-the-art performance on SR tasks. It has been applied to better capture user preference changes by learning the latent relationships between users and item sequences.

The promising performance of GNN for session-based recommendation is based on a large scale of sensitive data collected from user devices, which raises regulatory challenges for modern recommendation systems to deliver secure and privacy-preserving services. In addition, building efficient and scalable GNNs on large-scale data is challenging. The recent emerging federated learning (FL) is a candidate paradigm for recommendation systems that build models without sharing local data on devices. Furthermore, it naturally provides the personalization ability to the devices by fine-tuning the distributed models using local data. More importantly, applying FL on existing GNNs for session-based recommendation can easily solve scalability issues without building an ad hoc GNN model for large-scale data. In the FL scheme, only dozens of data samples are processed and used to train local models in devices, which can be done in much shorter time with lower computation costs. In short, the FL scheme is urgent under large-scale data for privacy-preserving in session-based recommendations with GNNs. In the rest of this paper, we use *GNN-SR* as the abbreviation for session-based recommendation with GNNs.

**Fig. 1:**
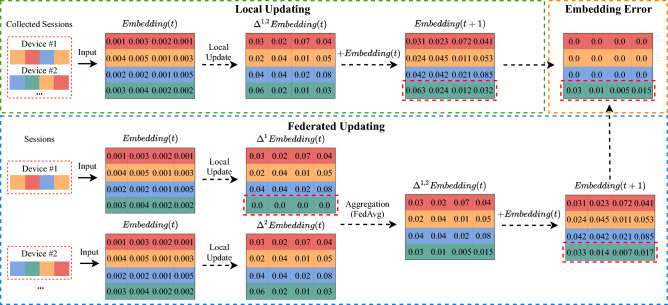
Incorrect aggregation of graph embedding when applying FL for GNN-SR.

However, applying GNNs for such a scenario with FL is still challenging. Firstly, constructing the global graph embedding in a centralized manner is forbidden due to the privacy-preserving constraints of FL. An alternative solution is to aggregate encoded local graph embedding constructed with local sessions. However, two-fold challenges lay ahead: 1) local graphs in devices contain minimal relation information, causing inefficient merging of these graphs. 2) There exists an aggregation error issue when directly aggregating local graph embedding. We illustrate this issue in Figure 1. The “local updating” scheme is training a GNN model in a centralized manner, which is supposed to be “correct.” In the “federated updating” scheme, the distributed devices collaboratively train models, and a central server aggregates graph embedding from different devices using FedAvg^23^. The embedding matrix in “embedding error” is the difference between the correct and the aggregated embedding. The conventional FedAvg fails to aggregate the graph embedding correctly, since the weights of rarely clicked items (i.e. long-tail items that are only clicked in a small part of devices) are incorrectly divided by the number of devices. However, it is not permitted to observe who and how many users have clicked these items due to the privacy-preserving constraints of federated learning.

Another fundamental challenge is the performance degradation caused by the extreme non-IIDness (non-Independent and Identically Distributed) in real-world, large-scale recommender systems. The user preferences usually diverge a lot and are probably distributed in a power-law tail. Although FL was intentionally designed for such non-IIDness, it still suffers a significant loss in performance when local data in devices differ significantly from each other. To temper the negative impact of non-IIDness, some adaptive methods for FL are proposed^24,25^. These methods adopt the well-known adaptive methods to optimize deep learning models in FL to improve generalization. However, existing adaptive methods may choose aggressive learning rates under extreme non-IID cases in the FL context, which may poison the performance. Putting the above issues together, the errors when applying FL in GNN-SR are easily magnified. Thus, efficient federated learning methods for these issues are needed.

This paper proposes a federated learning framework for GNN-SR to address the issues mentioned above. Our contributions are summarized as follows:

- We propose FedGA (**Fed**erated **G**raph **A**ggregation), a novel adaptive federated optimization method to alleviate performance issues caused by incorrect embedding updating and extreme non-IIDness.
- Two distinct strategies in FedGA, namely **D**ivergence-**R**esistant **A**ggregation (DRA) and **C**onditional **S**econd-**M**oment **E**stimation (C-SME) to eliminate aggressive learning rates in existing adaptive FL methods and hence improve model performance.
- We analyze the FedGA’s convergence bound theoretically and obtain the same results as the best-known adaptive methods.
- We conduct extensive experiments on large-scale SR datasets to validate our framework’s efficiency under different levels of non-IIDness. The results indicate that our approach outperforms other adaptive FL methods and retains similar performance to locally trained GNN-SR models.

## Related work

In this section, we review and compare related GNN-SR methods, FL methods for non-IID data, adaptive FL methods, and FL methods for GNN models.

**Session-based Recommendation using GNNs**. Incorporating GNNs has become an efficient practice for session-based recommendations. Such methods model the item sequence in a session as a graph structure or embedding to capture items’ latent relationships in different sessions. Xu et al.^14^ proposed a Graph Contextualized Self-attention (GC-SAN), which combines a self-attention network and GNN to enhance the performance of the recommendation. Wu et al.^26^ converted sequence data of items to a structured direct graph and increased the weights of the latest item, which finally improved the representation of the latest user interests. Qiu et al.^15^ proposed a Full Graph Neural Network (FGNN), an end-to-end model for the next-item recommendation in sessions. The main idea of this work is to learn the inherent order of the transition pattern of items. They applied 1) a multiple Weighted Graph Attention (WGAT) layer network to learn and assign appropriate weights to different neighbors in a graph and 2) a read-out function to generate graph-level representation. Song et al.^16^ proposed a dynamic graph attention neural network to model dynamic user behaviors and infer influencers according to the context of user interest. Yu et al.^17^ proposed the Target Attentive Graph Neural Network (TAGNN), which captures complex item transitions in sessions. Qiu et al.^18^ proposed the Global Attributed Graph (GAG), which combines user embedding and a long-term session sequence as the global attribute to improve the performance on long-term user interests.

**Federated Learning Methods for non-IID Data**. Alleviating the impact of non-IID data across large-scale devices is one of the key tasks for FL. McMahan et al. proposed FedAvg, the first method that claims promising performance for collaborative and privacy-preserving machine learning on non-IID data^23^. However, recent studies have observed unstable performance when the data is high-level non-IID. Zhao et al.^27 ^discovered a 55% performance degradation for FedAvg with highly skewed non-IID data. They argued that the degradation is caused by the divergence of model weights and the calculated weight divergence between local models using Earth-Mover Distance (EMD). However, their method requires globally shared data, which usually violates the data-sharing constraints in FL systems. Xie et al.^28^ proposed a method using multiple centers to better capture the pattern of non-IID data. Briggs et al.^29^ proposed a clustering approach that evaluates the similarities of different device models and groups them for a more robust aggregation. Yu et al.^30 ^proposed three strategies to improve performance on non-IID data. However, the above methods did not provide theoretical convergence guarantees. Li et al.^31^ gave the first theoretical convergence bounds of FedAvg on non-IID data. Nevertheless, its convergence ability is not proven for non-convex problems. Sahu et al.^32^ proposed FedProx, a FedAvg variant that applies an $L_2$ regularizer term in the objective functions of local models, and proved convergence guarantees for both convex and non-convex problems. Similarly, Shoham^33^ proposed Federated Curvature, which introduces a penalty term to reduce the impact of non-IIDness. Jeong et al.^34^ proposed federated augmentation (FAug), a data augment method that collaboratively trains a generative model in devices to generate more local data in devices. The results show a significant improvement in the accuracy of FedAvg with augmented data. Cong et al.^35^ introduced a federated learning method that relies on a greedy strategy. This strategy gradually searches for models that are partially optimal and combines them to achieve the global model. However, the above FL methods do not apply to both GNNs and session-based recommendations.

**Adaptive Federated Optimization**. Inspired by successful adaptive optimization methods, Reddi et al.^25^ proposed a federated version of adaptive optimization methods, namely FedAdagrad, FedAdam, and FedYogi. Furthermore, they analyzed and provided convergence guarantees for non-convex problems. However, in the session-based recommendation, the above adaptive methods may suffer from the fluctuate adaptive learning rates due to the extreme non-IIDness, resulting in unstable performance. Ju et al.^36^ proposed a federated learning method named AdaFedAdam, which addresses the fairness problem by formulating it as a multi-objective optimization problem, analyzes the performance of Adam optimizer, and proposes an adaptive approach to achieve fair and efficient federated learning with enhanced global model performance. Finally, some adaptive methods fail to obtain stable performance, as the server cannot observe the data size in distributed devices, causing incorrect aggregation on graph embedding.

**Federated Learning with GNNs**. Jiang et al.^37^ proposed Feddy, a distributed and secured framework to learn object representations from multi-user graph sequences. However, Feddy requires full participation of all distributed nodes, computes all adjacent information from these nodes, and records the status of the node for each second, which is not efficient and practical in large-scale real-world recommender systems. Sajadmanesh and Gatic-Perez^38^ developed a GNN learning algorithm that preserves privacy with formal privacy guarantees based on Local Differential Privacy (LDP) to protect node characteristics. However, their method is still under full-node participation and constructs a global graph using all nodes, which is forbidden in common FL scenarios since all nodes are isolated. Zheng et al.^39^ proposed ASFGNN (Automated Separated-Federated Graph Neural Network) for GNN under non-IID data. However, ASFGNN uses only conventional averaging in model aggregation, which will eventually cause the information loss issue as we mentioned previously, and such an issue is not easy to solve by optimizing hyper-parameters. In addition, ASFGNN uses Bayesian Optimization (BO) to find the best hyperparameters, which is a black-box process, and the performance may not be stable in federated learning when model gradients vary. Wang et al.^40^ proposed two federated learning methods for GNNs under non-IID data. Their methods can generalize into new label domains thanks to self-training strategies. However, their methods have two-fold limitations: 1) They assume that each device has the complete graph in experiments, which is not realistic when the global graph contains a large scale of nodes. 2) They argue that the overall performance is greatly affected by the fraction of overlap between device graphs, which is commonly seen in real-world recommender systems. Wu et al.^41^proposed FedGNN, a federated GNN framework for privacy-preserving recommendations. The authors apply local differential privacy techniques to protect user information and user-item interactions. However, although encrypted user embedding is aggregated in FedGNN, the information loss issue in aggregating graph embedding was not addressed. Wan et al.^42^ proposed a prototype-based approach to tackle the domain shift in federated graph learning. Their method learns generalizable prototypes across clients to align local and global representations, thus improving generalization under distributional heterogeneity. To date, there is no effective federated learning framework for GNNs that directly addresses challenges in session-based recommendation or the embedding aggregation issues specific to GNN-SR under non-IID conditions.

Compared to existing methods, our method does not hold the assumption that each device shall hold the same global graph, and it is flexible to realistic recommender systems under extreme non-IID data.

## Preliminaries

In this paper, we focus on federated learning in *cross-device* settings for GNN-SR. In this section, we give the preliminary definitions of FL and GNN-SR.

**Session-based Recommendation with GNN**. We use similar definitions proposed by Wu et al.^26^ as a typical example of GNN-SR. Let $mathscr{V}=\{\nu _1, \nu _2, ..., \nu _n\}$ be the item set in all sessions, where *n* is the total number of items. We denote a user session as $s=\{\nu _{s,1}, \nu _{s,2}, ..., \nu _{s,n_s}\}$, where $n_s$ is the number of items in session *s*, and $\nu _{s,i}\in mathscr{V}$. We then define the graph structure of the session *s* as $mathscr{G}_s=(mathscr{V}_s, mathscr{E}_s)$, where $mathscr{V}_s$ is the sequence of clicked items, and $mathscr{E}_s$ is the edge that links two consecutive clicked items in $mathscr{V}_s$. The session graphs are then converted to the embedding as the input of a GNN model. Finally, the GNN model learns the latent session embedding vectors and predicts the top-*k* items with the highest probability as the next recommended items. Please note that in different GNN-SR methods, the graph structure and the construction of graph embedding can be different. For example, TAGNN introduces the adjacency matrix $mathscr{A}$ in the graph structure of sessions, i.e., $mathscr{G}_s=(mathscr{V}_s, mathscr{E}_s, mathscr{A}_s)$, and introduces pseudo-interaction items in the graph embedding.

**Federated Learning**. Federated learning is minimizing the objective function of a global model that can be described as:

1 $$\begin{aligned} \min _{w\in \mathbb {R}^d} f(w) = \frac{1}{m}\sum _{i=1}^{m}F_i(w), \end{aligned}$$

where $F_{i}(w)$ is the objective function of the local model in $i^{th}$ device, *m* is the total number of devices, and *d* is the model dimension.

**Federated Learning on GNN-SR**. The typical GNN models for session-based recommendation include the graph embedding layer and the model weights layers (e.g.,^17,26^). The device models learn and update the weights of the embedding layer and other middle layers locally using only local data, and then send the model parameters (including the embedding layer and middle layers) to the central server. The central server aggregates the collected model gradients using federated averaging:

2 $$\begin{aligned} w^{t+1} = \frac{1}{m}\sum _{i=1}^{m}w_i^{t} = w^t + \frac{1}{m} \sum _{i=1}^{m}\left( w_i^{t} - w^{t}\right) = w^t + \frac{1}{m} \sum _{i=1}^{m}\Delta w_i^t, \end{aligned}$$

**Fig. 2:**
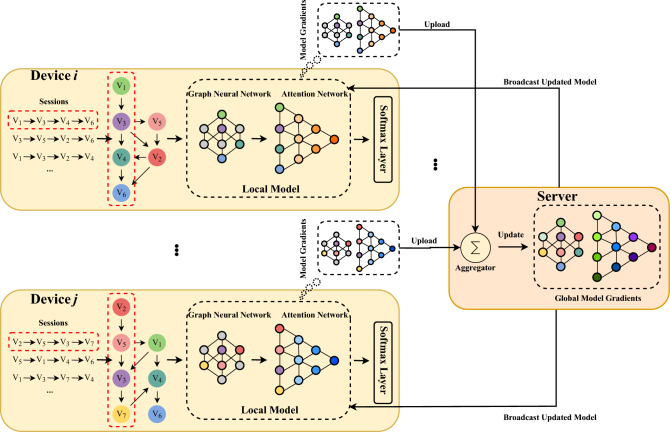
An example of GNN-SR under the federated learning scheme. We use the GNN model proposed in Wu et al.^26^ as the example GNN model.

where $w_i^t$ includes model parameters of both the embedding layer and middle layers as mentioned earlier.

An intuitive way to train GNN models collaboratively using FL is as follows: 1) building the embedding session locally on devices, 2) training local GNN models using the embedding of the local session, and 3) updating the global model using the parameters collected from the GNN model on the central server. We give an example of GNN-SR under FL in Figure 2. However, as discussed earlier, directly updating the global model using conventional FL (e.g. FedAvg) may result in performance degradation. The current emerging adaptive federated optimization (for example, FedAdam and FedYogi proposed in Reddi et al.^25^) is a candidate solution for GNN-SR due to adaptive strategies in updating the global model. In the next section, we propose FedGA, a novel adaptive FL method for GNN-SR.

## The proposed method

### Method overview

We illustrate the main FedGA processes in Algorithms 1 and 2. The central server initializes a neural network model as the global model, sets the maximum number of global epochs *T*, and initializes the current epoch number $t=0$. At each global epoch, the central server randomly samples part of the device set $S_t$ from the devices. The sampled devices initialize their local models using the received global model and train them using the corresponding local data. When local training is finished, the sampled devices upload trained model gradients to the central server. Then, the central server calculates the mean of the collected model gradients $\Delta _t$ (lines 8 and 9 of Algorithm 1).

**Algorithm 1:**
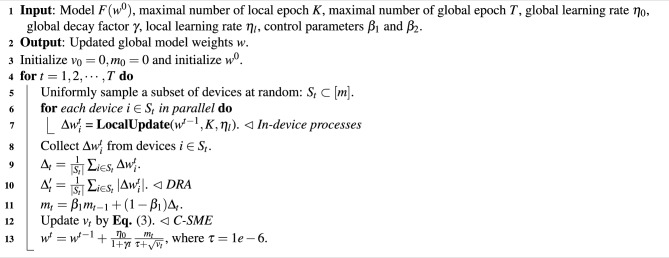
FedGA - Server

**Algorithm 2:**
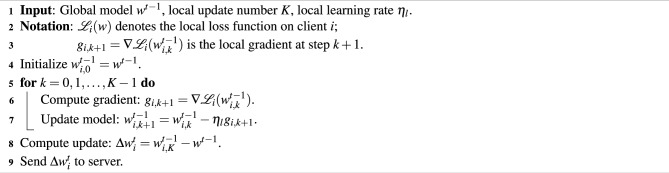
LocalUpdate

The rest of the processes are similar to the procedures of adaptive optimization, i.e., calculating the first- and second-moment estimations of model gradients and using them to update the global model. Firstly, for the first-moment estimation, we introduce Divergence-Resistant Aggregate (DRA) to alleviate the impact of sparse gradients collected from devices (line 10 of Algorithm 1). For the second-moment estimation (SME), we propose Conditional Second Moment Estimation (C-SME), which carefully estimates the second-order moments under three conditions, i.e.,

3 $$\begin{aligned}&v_t = \left\{ \begin{aligned}&v_{t-1} + (1 - \beta _2)\sqrt{({\Delta '_t}^2 + v_{t-1})({\Delta '_t}^2 - v_{t-1})}&\text { (Case 1)} \\&v_{t-1}-(1 - \beta _2)\sqrt{(v_{t-1} - {\Delta '_t}^2)(3{\Delta '_t}^2 - v_{t-1})}&\text { (Case 2)}\\&v_{t-1}-(1 - \beta _2){\Delta '_t}^2&\text { (Case 3)} \end{aligned} \right. \end{aligned}$$

where Case 1, Case 2 and Case 3 represent conditions $v_{t-1} \le {\Delta '_t}^2, {\Delta '_t}^2 < v_{t-1} \le 2{\Delta '_t}^2$ and $v_{t-1}> 2{\Delta '_t}^2$ respectively. Case 1, Case 2, and Case 3 respectively correspond to: 1) relatively uniform client behavior and activity levels, where inter-client gradient differences are small (i.e., low variance); 2) moderately sparse and divergent scenarios, where user behavior starts to differ across clients; and 3) extremely sparse or highly non-IID settings, such as when some clients have almost no interactions (e.g., long-tail users), resulting in large gradient magnitude disparities across devices.

Please note that the additional server-side operations from DRA and C-SME are lightweight. DRA requires only simple element-wise operations for averaging absolute gradients, and C-SME involves conditional updates with basic arithmetic. These introduce negligible overhead compared to the cost of local training and do not affect server scalability or latency. We will explain the above three cases in Section 9. Finally, the central server updates the global model using the results of DRA and C-SME.

### Divergence-Resistant Aggregation (DRA)

The motivation for divergence-resistant aggregation is alleviating extreme non-IIDness. In session-based recommendation, the models in the devices produce sparse large-scale gradients, which can magnify the performance of the aggregated model. In adaptive federated optimization methods, aggregating sparse models will significantly decrease the denominator part (line 13 of Algorithm 1), i.e., $\sqrt{v_{t}} + \tau = \sqrt{\beta _2 v_{t-1} + (1 - \beta _2) \Delta _t} + \tau$, where $\tau$ is a nonzero number that avoids dividing by zero. Thus, in such a case, the global model is updated in a more aggressive step size, causing fluctuated model performance. To reduce aggressive updating, we additionally compute the averaging of model gradients’ absolute values as $\Delta ^{'}_t$ (line 10 of Algorithm 1), thus keeping the denominator part bigger to smooth the overall step size, which alleviates the vibration of model performance.

Although DRA addresses the sparsity-induced instability in federated model aggregation, particularly from long-tail clients whose average $|\Delta w|$ may be extremely small, it alone cannot fully guarantee training stability. In such cases, even with inflated denominators, sharp variance across devices can still lead to unstable updates. To further mitigate this, we introduce the Conditional Second Moment Estimation (C-SME) mechanism, which explicitly accounts for the update patterns based on the magnitude of $|\Delta w|$ and adjusts $v_t$ accordingly in three well-designed cases. The next section elaborates on how the C-SME stabilizes the training even when $|\Delta w|$ is near zero or varies sharply.

### Conditional Second-Moment Estimation (C-SME)

To better illustrate the motivation behind C-SME and its advantages over existing strategies, we first revisit the second-moment estimation (SME) mechanisms adopted in existing adaptive federated optimization methods. FedAdam^25^ uses the SME formulation originally proposed in Adam^43^:

4 $$\begin{aligned} v_t = \beta _2 v_{t-1} + (1-\beta _2) {\Delta '_t}^2 = v_{t-1} - (1- \beta _2) (v_{t-1} - {\Delta '_t}^2), \end{aligned}$$

which performs a weighted average between the historical second moment $v_{t-1}$ and the squared mean gradient ${\Delta '_t}^2$. FedYogi modifies this update with a sign operator to suppress aggressive changes:

5 $$\begin{aligned} v_t = v_{t-1} - (1-\beta _2) {\Delta '_t}^2 \cdot \text {sign}(v_{t-1} - {\Delta '_t}^2). \end{aligned}$$

Although both strategies aim to stabilize the learning rate by adapting the second moment, they exhibit undesirable behaviors under federated training conditions in the real world. Specifically, in highly non-IID settings with sparse or divergent client updates, both methods can cause sudden or overly aggressive changes to $v_t$, harming convergence stability. To systematically study this issue, we define a ratio variable $V_t$ as:

6 $$\begin{aligned} V_t = \frac{v_{t-1}}{{\Delta '_t}^2}, \end{aligned}$$

which captures the relative scale between the historical second moment and the current aggregated gradient magnitude. We also define a normalized change term:

7 $$\begin{aligned} \Delta \tilde{v}_t = \frac{v_t - v_{t-1}}{-(1-\beta _2){\Delta '_t}^2}, \end{aligned}$$

so that $\Delta \tilde{v}_t$ can be directly interpreted as the scaled change rate of $v_t$.

**Fig. 3:**
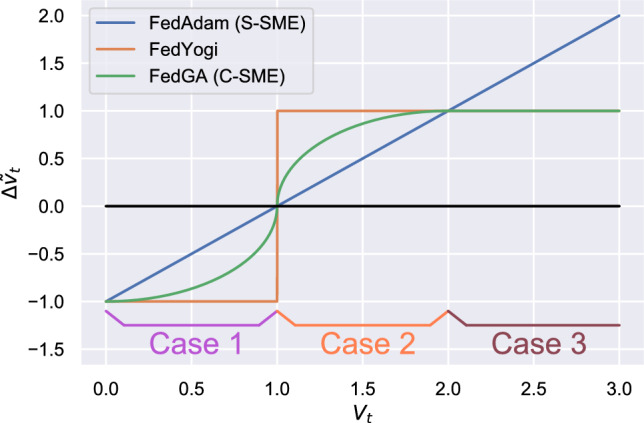
Interactions between $V_{t-1}$ and $\Delta \tilde{v}_{t}$.

Ideally, the value of $\Delta \tilde{v}_t$ should change smoothly with respect to $V_t$ during training. However, Figure 3 shows that this is not the case. In FedAdam, $\Delta \tilde{v}_t$ grows linearly with $V_t$, leading to aggressive updates when $V_t$ becomes large. In FedYogi, the change is bounded in magnitude, but a discontinuity occurs at $V_t=1$, where $\Delta \tilde{v}_t$ jumps from $-1$ to 1. This sudden flip introduces instability during the transition from Case 1 to Case 2. To address these limitations, we propose a continuous, case-based update mechanism in C-SME in Eq. (3). This design ensures a smooth transition across different regions and adapts to the degree of gradient divergence. The three cases in C-SME are defined as:

- **Case 1** ($v_{t-1} \le {\Delta '_t}^2$): The variance of aggregated gradients is relatively high and the client behaviors are similar and dense. We apply a gentle increase to $v_t$ to support learning without overshooting.
- **Case 2** (${\Delta '_t}^2 < v_{t-1} \le 2{\Delta '_t}^2$): A transitional region where gradient sparsity or divergence begins to appear across clients. We apply a smooth decay to avoid the sharp jump seen in FedYogi.
- **Case 3** ($v_{t-1}> 2{\Delta '_t}^2$): A highly non-IID regime where gradient magnitudes vary significantly due to client heterogeneity (e.g., long-tail users with few interactions). Conservative fixed-rate decay is used to avoid instability.

These three cases correspond to common scenarios in federated recommendation: Case 1 represents homogeneous active clients; Case 2 captures moderate divergence; and Case 3 reflects severely sparse and skewed user behavior. By tailoring the second-moment update in each case, C-SME ensures that $v_t$ evolves continuously and smoothly throughout the training, which in turn stabilizes the effective learning rate in FedGA.

Finally, we note that the server-side computation introduced by C-SME is lightweight and involves only basic arithmetic and conditional logic. This imposes negligible overhead compared to local training or communication costs.

### Theoretical analysis

#### Overview

The goal of this section is to establish the convergence guarantee of FedGA (Corollary 1). The key challenge in federated GNN-based recommendation lies in handling sparse and divergent gradients across clients, especially when local data distributions vary significantly. To analyze this, we start by formalizing the standard federated optimization problem and modeling the local training dynamics across devices. We make a set of standard assumptions (ie, unbiased stochastic gradients, bounded smoothness, bounded gradients, and bounded gradient variance) to allow tractable analysis. Building on these, we decompose the global gradient update into the average of local updates and then analyze how the divergence in local models affects the global optimization trajectory. The derivation focuses on bounding the norm of the gradient of the global objective, $\Vert \nabla f(x_t)\Vert ^2$, over training rounds. We derive an upper bound that depends on the local update steps, the variance between clients, and the adaptive learning dynamics introduced by FedGA (notably the conditional second-moment estimation). The result shows that FedGA achieves a convergence rate of $mathscr{O}(1/\sqrt{T})$, which matches the best-known rates for adaptive federated optimization.

#### Analysis details

We denote $x \in \mathbb {R}^d$ and $y \in \mathbb {R}^d$ as any two vectors in $\mathbb {R}^d$ since these results are satisfying for all $x \in \mathbb {R}^d$. Certainly, the model weights $w \in \mathbb {R}^d$ are also satisfying in these theories. First, we propose the assumptions.

##### Assumption 1

(Unbiased Local Gradient) The $i^{th}$ device’s local stochastic gradient $g_i(x)$ is unbiased estimation of subgradient $\nabla F_i(x)$, i.e., $\mathbb {E}_{z\in mathscr{D}_i}\left[ g_i(x)\right] = \nabla F_i(x)$.

##### Assumption 2

The local function $F_i$ is L-smooth for all $i\in [m]$, i.e., $\Vert \nabla F_i(y) - \nabla F_i(x)\Vert \le L\Vert x-y\Vert$, and $F_i(y) \le F_i(x) + \langle \nabla F_i(x), y-x\rangle + \frac{L}{2}\Vert x-y\Vert ^2$.

##### Assumption 3

The local objective function $f_i(x,z)$ has *G*-bounded gradients, that is, for any $i\in [m]$, $x\in \mathbb {R}^d$ and $z\sim mathscr{D}_i$, we have $\Vert \left[ \nabla f_i(x,z)\right] _j\Vert \le G$ ($G>0$), for all $j\in [d]$.

##### Assumption 4

**Locally**, the local objective functions $f_i(x,z)$ have a $\sigma _l$-bounded variance, that is, $\mathbb {E}\left[ \left| \left[ \nabla f_i(x,z)\right] _j - \left[ \nabla F_i(x)\right] _j\right| ^2\right] \le \sigma _{l,j}^2$ for all $x\in \mathbb {R}^d$, $i\in [m]$, and $j\in [d]$. **Globally**, the global variance is bounded, i.e.,

$\frac{1}{m}\sum _{i=1}^{m}\left[ \left[ \nabla F_i(x)\right] _j - \left[ \nabla f(x)\right] _j \right] ^2 \le \sigma _{g,j}^2$ for all $x \in \mathbb {R}^d$ and $j\in [d]$.

##### Theorem 1

Let Assumption 1 to 4 hold and with proper local stepsize $\eta _l\le \frac{2}{KL\sqrt{43}}$. Let $\eta _l$ and $\eta$ be the local step size and the global step size, respectively. The iterations of FedGA satisfies

$$\begin{aligned}&\min _{t} \Vert \nabla f(x_t)\Vert ^2 \le \frac{2(\sqrt{\beta _2}\eta _l K G+\tau )}{T\eta \eta _l K}(f(x_{0}) - \mathbb {E}_{t}[f(x_{T})])\\&+ \frac{2(\sqrt{\beta _2}\eta _l K G+\tau )}{\tau ^2} \Biggl \{{2\eta L\eta _l K}\sigma _l^2 + 4\sqrt{1-\beta _2}G\eta _l K[\sigma _l^2+ \sigma _g^2 + d G^2 ] \\&+ {36\eta \eta _l^3 K^3 L^4 + \sqrt{1-\beta _2} 72 G\eta _l^3 K^3 L^2 + 9\eta _l^2 K^3 L^2\tau + \eta \eta _l LK^2}{dG^2} \\&+ {6\eta \eta _l^3 K^2 L^4+ 12 \sqrt{1-\beta _2} G\eta _l^3 K^2 L^2 + \frac{3\eta _l^2 K \tau L^2}{2}}\sigma ^2 \Biggr \} \end{aligned}$$

##### Corollary 1

Setting $\eta _l=\min \{\frac{1}{\sqrt{T}},\frac{2}{KL\sqrt{43}}\}$ and $\eta = \frac{1}{\sqrt{T}}$, then we have

$$\begin{aligned} \min _{0\le t\le T-1} \Vert \nabla f(x_{t})\Vert ^2 \le mathscr{O}\left( \frac{1}{\sqrt{T}} \right) \end{aligned}$$

The above theorem and corollary provide the theoretical convergence bound of FedGA. The results indicate that FedGA obtains a convergence speed $mathscr{O}(1/\sqrt{T})$ similar to existing adaptive federated optimization methods.

## Experiments

We conducted empirical experiments to answer the following research questions (RQ).

- **RQ1**: Can FedGA outperform the state-of-the-art adaptive FL methods on GNN-SR?
- **RQ2**: How does each strategy contribute to the performance of FedGA?

We first describe the datasets and settings and then analyze the experiment results to answer the above research questions.

### Datasets

We test the performance of FedGA on four open datasets, namely Gowalla, LastFM, Yoochoose (1/4 and 1/64), and Retailrocket. Table 1 shows the statistics of the datasets mentioned above. In addition to the statistics, we also introduce a novel metric that describes the degree of non-IIDness based on the distribution of Jaccard distances between instances. Specifically, the metric is defined as

8 $$\begin{aligned} JD = \frac{1}{m}\frac{1}{m-1}\sum _{i=1}^{m}\sum _{j=1,j{\not =}i}^{m}{(1-\frac{|mathscr{I}_{(i)} \cap mathscr{I}_{(j)}|}{|mathscr{I}_{(i)} \cup mathscr{I}_{(j)}|})}, \end{aligned}$$

where $mathscr{I}_i$ is the set of items in device *i*. The higher JD indicates a higher degree of non-IIDness. Next, we illustrate these datasets in detail.

**Table 1:** Statistics of session-based recommendation datasets.

Dataset	#Click	#Device	#Item	#Sess.(train)	#Sess.(test)	Avg. Len	Avg. #Sess.	Max #Sess.	Min #Sess.	#Sess. Std.	#Skewness	#Kurtosis	Max #Item	Min #Item	#Item Std.	JD
Gowalla	6, 442, 890	47, 194	29, 814	688, 561	77, 122	2.32	14.59	999	1	39.63	8.68	113.93	359	1	19.03	0.89
LastFM	3, 804, 922	981	36, 949	1, 238, 523	137, 646	5.70	1262.51	8, 826	2	1188.08	1.53	3.36	1968	2	252.99	0.56
Yoochoose1/4	9, 310, 403	1, 991, 564	37, 484	6, 153, 933	55, 898	4.72	3.09	199	1	4.06	7.28	128.21	196	1	3.26	0.84
Yoochoose1/64	2, 315, 442	124, 472	37, 484	394, 577	55, 898	5.14	3.17	145	1	4.37	6.83	96.27	139	1	3.52	0.83
Retailrocket	893, 945	318, 697	76, 061	799, 930	87, 512	3.11	2.51	18	1	2.61	2.79	9.20	19	1	1.72	0.94

- **Gowalla**^44^: Gowalla is a point-of-interest real-world dataset collected from a social network for users’ check-in. We follow the same data processing settings as proposed in Guo et al.^10^ We selected the 3, 000 most popular places and defined a session as a user’s check-in in one day. Finally, we dropped sessions that contained more than 20 check-ins or less than 2.
- LastFM-1K (LastFM-1K: http://ocelma.net/MusicRecommendationDataset/lastfm-1K.html): LastFM is a popular music recommendation dataset that contains user clicks (user, time, artist, and song) collected between 2004 and 2009. We followed the same pre-processing as in^13^. We selected the most popular 40,000 artists and split each session as the clicks in every 8-hour interval for each user. We dropped sessions that were longer than 20 or less than 2.
- Yoochoose (Yoochoose dataset: http://2015.recsyschallenge.com/challege.html): Yoochoose is a recommendation dataset published in the 2015 Recsys challenge that contains six-month click streams on an e-commerce website. We followed the same data processing as proposed in^26^. We used the click streams on the last day as the validation set and selected the last 1/4 and 1/64 in the rest of the data as two training sets (Yoochoose 1/4 and 1/64). We dropped the items that are clicked with less than 5 users and sessions that contain less than 2 items.
- Retailrocket (Retailrocket dataset: https://www.kaggle.com/retailrocket/ecommerce-dataset): Retailrocket is an E-business recommendation dataset that contains 6-month click streams of users. We followed the same pre-processing procedures as proposed in^14^. We dropped the items that are clicked by less than 5 users and the sessions that are longer than 20 or less than 2.

We split the training data and the validation data for the above datasets from the original sessions. Specifically, an original session sequence of user *i* can be defined as $s_i=\{\nu _{{s_i},1}, \nu _{{s_i},2}, \nu _{{s_i},3},..., \nu _{{s_i},n_i}\}$, then its training set and validation set are processed as tuples that contain a session sequence and a label, e.g., $(\{\nu _{{s_i},1}\}, \nu _{{s_i},2})$, $(\{\nu _{{s_i},1}, \nu _{{s_i},2}\}, \nu _{{s_i},3})$, $(\{\nu _{{s_i},1}, \nu _{{s_i},2}, \nu _{{s_i},3}\}, \nu _{{s_i},4})$,..., $(\{\nu _{{s_i},1}, \nu _{{s_i},2}, \nu _{{s_i},3}, ..., \nu _{{s_i},n_i-1}\}, \nu _{{s_i},n_i})$.

### Baselines and metrics

To evaluate the performance of the proposed method, we compare FedGA with the following related federated learning methods:

- **FedAvg**^23^: one of the first federated learning methods that applies federated averaging to aggregate the model parameters collected from sampled devices. To date, it is still a simple but effective baseline for FL.
- **FedProx**^45^: FedProx is a modified version of FedAvg that introduces the $L_2$ regularization term in each client’s local objective, penalizing divergence from the global model.
- **FedAdam**^25^: state-of-the-art adaptive federated optimization method inspired by adaptive optimization methods. Shows promising performance on non-IID datasets.
- **FedYogi**^25^: a variant federated optimization method in which the SME part is defined in Eq.(5).

We validated the above federated learning methods on two GNN models for session recommendation:

- **SR-GNN**^26^ (Session-based Recommendation with Graph Neural Networks) models session sequences as structured graph data. GNN can capture complex transitions of items based on the session graph, which are difficult to reveal by previous conventional sequential methods. Each session is then represented as the composition of the global preference and the current interest of that session using an attention neural network.
- **TA-GNN**^17^ (Target Attentive Graph Neural Network) captures dynamic item transitions in user sessions and generates different session embedding for each target item. It introduces a pseudo-interacted item sampling technique to protect user privacy and a graph expansion method exploiting high-order user-item interactions.

All the methods were implemented using Python 3.7 and Pytorch 2.2. We ran the experiments on two machines with 8-core 2.8-GHz Intel CPUs and 64GB memory. We tuned the best hyperparameters for the above FL and backbone GNN models by performing a grid search. We ran each experiment 3 times and reported the mean values and standard deviations of the following metrics.

- **HR**@10/20 (Hit Rate): evaluates the accuracy of the model by calculating the proportion of correctly recommended items in the top 10/20 item list.
- **MRR**@10/20 (Mean Reciprocal Rank): it evaluates the model ranking capability by calculating the mean reciprocal ranks of the correctly recommended items. The value of MRR is 0 when the rank exceeds 10/20.

**Table 2:** Evaluation Results of FL Methods on SR-GNN and TA-GNN – Top@20 HR and MRR.

**HR@20**					
Methods	Yoochoose 1/64	Yoochoose 1/4	Gowalla	LastFM	Retailrocket
*SR-GNN*					
SR-GNN (Centralized)	$70.32 \pm 0.08$	$71.00 \pm 0.16$	$63.58 \pm 0.02$	$30.66 \pm 0.22$	$65.49 \pm 0.31$
FedAvg	$57.74 \pm 0.03$	$27.81 \pm 0.80$	$21.97 \pm 0.15$	$12.50 \pm 0.03$	$46.38 \pm 0.10$
FedProx	$57.32 \pm 0.04$	$27.60 \pm 0.75$	$21.65 \pm 0.13$	$12.18 \pm 0.06$	$46.14 \pm 0.11$
FedAdam	$65.87 \pm 0.27$	$58.55 \pm 1.04$	$51.30 \pm 0.18$	$27.39 \pm 0.15$	$51.05 \pm 0.17$
FedYogi	$69.15 \pm 0.19$	$67.04 \pm 0.24$	$57.71 \pm 0.12$	$27.82 \pm 0.11$	$61.00 \pm 0.09$
FedGA	$$\varvec{69.72}$$$$\varvec{\pm }$$$$\varvec{0.12}$$	$$\varvec{67.67}$$$$\varvec{\pm }$$$$\varvec{0.13}$$	$$\varvec{58.57}$$$$\varvec{\pm }$$$$\varvec{0.16}$$	$$\varvec{27.96}$$$$\varvec{\pm }$$$$\varvec{0.12}$$	$$\varvec{61.74}$$$$\varvec{\pm }$$$$\varvec{0.10}$$

*TA-GNN*					
TA-GNN (Centralized)	$70.87 \pm 0.22$	$71.43 \pm 0.10$	$63.28 \pm 0.05$	$31.12 \pm 0.10$	$65.74 \pm 0.08$
FedAvg	$29.51 \pm 0.33$	$28.13 \pm 0.50$	$22.17 \pm 0.10$	$12.23 \pm 0.05$	$44.29 \pm 0.09$
FedProx	$29.46 \pm 0.29$	$27.96 \pm 0.45$	$21.85 \pm 0.11$	$11.92 \pm 0.07$	$43.95 \pm 0.11$
FedAdam	$65.98 \pm 0.25$	$59.37 \pm 0.30$	$50.92 \pm 0.12$	$27.19 \pm 0.08$	$53.23 \pm 0.14$
FedYogi	$68.91 \pm 0.08$	$67.06 \pm 0.12$	$57.38 \pm 0.10$	$27.50 \pm 0.09$	$61.42 \pm 0.10$
FedGA	$$\varvec{69.17}$$$$\varvec{\pm }$$$$\varvec{0.05}$$	$$\varvec{67.20}$$$$\varvec{\pm }$$$$\varvec{0.08}$$	$$\varvec{58.00}$$$$\varvec{\pm }$$$$\varvec{0.07}$$	$$\varvec{27.60}$$$$\varvec{\pm }$$$$\varvec{0.06}$$	$$\varvec{61.47}$$$$\varvec{\pm }$$$$\varvec{0.03}$$

**MRR@20**					
Methods	Yoochoose 1/64	Yoochoose 1/4	Gowalla	LastFM	Retailrocket
*SR-GNN*					
SR-GNN (Centralized)	$30.10 \pm 0.05$	$31.49 \pm 0.15$	$31.93 \pm 0.07$	$11.04 \pm 0.02$	$41.47 \pm 0.07$
FedAvg	$25.98 \pm 0.02$	$17.84 \pm 0.36$	$11.61 \pm 0.05$	$5.76 \pm 0.03$	$39.52 \pm 0.02$
FedProx	$25.67 \pm 0.03$	$17.65 \pm 0.30$	$11.59 \pm 0.06$	$5.60 \pm 0.04$	$39.60 \pm 0.03$
FedAdam	$28.00 \pm 0.04$	$24.72 \pm 0.20$	$24.02 \pm 0.09$	$10.38 \pm 0.05$	$40.02 \pm 0.04$
FedYogi	$29.72 \pm 0.04$	$28.46 \pm 0.04$	$27.94 \pm 0.13$	$10.47 \pm 0.04$	$40.47 \pm 0.05$
FedGA	$$\varvec{29.75}$$$$\varvec{\pm }$$$$\varvec{0.07}$$	$$\varvec{28.67}$$$$\varvec{\pm }$$$$\varvec{0.01}$$	$$\varvec{28.61}$$$$\varvec{\pm }$$$$\varvec{0.07}$$	$$\varvec{10.55}$$$$\varvec{\pm }$$$$\varvec{0.03}$$	$$\varvec{40.92}$$$$\varvec{\pm }$$$$\varvec{0.01}$$

*TA-GNN*					
TA-GNN (Centralized)	$30.58 \pm 0.06$	$31.72 \pm 0.10$	$31.80 \pm 0.05$	$11.29 \pm 0.08$	$41.52 \pm 0.11$
FedAvg	$12.54 \pm 0.17$	$17.95 \pm 0.20$	$11.51 \pm 0.05$	$5.80 \pm 0.03$	$38.83 \pm 0.02$
FedProx	$12.47 \pm 0.15$	$17.71 \pm 0.19$	$11.32 \pm 0.06$	$5.51 \pm 0.04$	$38.54 \pm 0.03$
FedAdam	$28.07 \pm 0.17$	$24.81 \pm 0.18$	$24.26 \pm 0.09$	$10.38 \pm 0.05$	$39.36 \pm 0.11$
FedYogi	$30.02 \pm 0.17$	$28.53 \pm 0.12$	$27.90 \pm 0.13$	$10.41 \pm 0.07$	$41.07 \pm 0.13$
FedGA	$$\varvec{30.32}$$$$\varvec{\pm }$$$$\varvec{0.08}$$	$$\varvec{28.84}$$$$\varvec{\pm }$$$$\varvec{0.10}$$	$$\varvec{28.63}$$$$\varvec{\pm }$$$$\varvec{0.07}$$	$$\varvec{10.49}$$$$\varvec{\pm }$$$$\varvec{0.06}$$	$$\varvec{41.07}$$$$\varvec{\pm }$$$$\varvec{0.03}$$

**Fig. 4:**
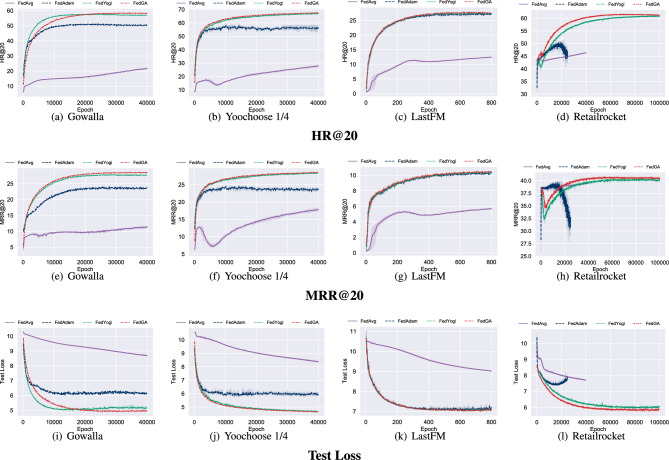
Performance of FedGA on four datasets. From top to bottom, rows show HR@20, MRR@20, and test Loss.

### Overall Performance (RQ1)

To answer RQ1, we compare the performance of FedGA with other baselines in session recommendation datasets. We show the results of different FL methods on SR-GNN and TA-GNN in Table 2. In summary, FedGA outperforms other baselines in all datasets and GNN models. Its performance is closer to the centralized trained GNN models compared to other FL baselines. Specifically, FedGA leads to a maximum relative improvement of 166. 59% in HR @ 20 and 146. 23% in MRR @ 20 over FedAvg in SR-GNN. Compared to FedYogi, FedGA improves HR@20 and MRR@20 by 1.49% and 2.4% in SR-GNN, respectively. The results show that our method performs well thanks to the proposed strategies. Although FedProx adds $L_2$ regularization to limit local model drift, it does not effectively reduce aggregation errors caused by client-specific embedding updates as we mentioned earlier, often performing slightly worse than FedAvg.

Figure 4 shows HR@20, MRR@20 and the test loss curves of federated learning methods. It can be seen that FedAvg fails to converge in all datasets. It should be noted that, though FedAdam achieves similar results in Table 2, FedAdam converges slower in Gowalla and Yoochoose, as shown in Figure 4. Moreover, on the two datasets with the highest JD (i.e., Retailrocket and Gowalla), FedAdam performs worse and is unstable. In Retailrocket, FedAdam experiences two performance crashes at the beginning of training and after 20,000 global epochs (see Figure 4(d), (h), and (i)). This phenomenon implies that FedAdam estimates the learning rates more aggressively, resulting in severe performance degradation. In contrast, FedYogi uses a conservative strategy to update learning rates, which makes the performance stable. Moreover, FedGA retains better accuracy and stability simultaneously by incorporating DRA and C-SME. As shown in Figure 4, FedGA reaches a comparable or better accuracy with fewer rounds, e.g., on Retailrock with high JD, FedGA reaches 60% HR@20 in less than 40,000 rounds versus more than 60,000 for FedYogi, reducing the total communication and training cost. The extra server-side computation introduced by DRA and C-SME is minimal and does not affect overall latency.

**Fig. 5:**
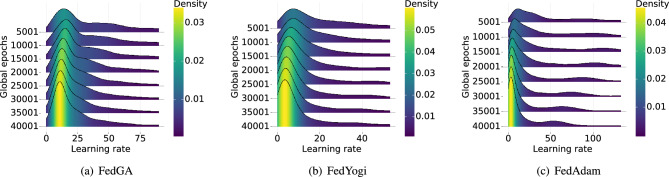
Learning rates distribution in different adaptive federated learning methods.

**Fig. 6:**
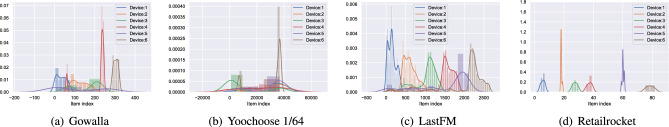
Item density in different devices.

To better understand the effects of the proposed strategies and differences between FedGA and other adaptive methods, we plot the learning rate distributions of FedGA, FedYogi, and FedAdam in Figure 5. The results clearly show that the learning rate smoothly distributes our proposal in all training epochs. Although the learning rate distributions of FedYogi and FedAdam are rather long-tailed, it implies that the variances of learning rates are higher, which may cause severe performance vibration, especially on higher-level non-IID data. In such circumstances, sessions vary on different devices (see Figure 6), leading to higher local model differences, consequently causing a more aggressive change in learning rates and, finally, affecting the stability of the global model. In FedGA, DRA and C-SME effectively control the distribution of learning rates during training, thus making the model more stable.

**Table 3:** Sensitivity analysis of FedGA on the Gowalla dataset. Left: varying local update steps $K$; Right: varying global learning rate $\eta _0$.

	HR@20	MRR@20	HR@10	MRR@10
(a) Effect of Local Update Steps K				
1	58.37	28.69	49.98	28.13
5	58.19	27.99	49.07	27.42
10	57.75	27.9	48.96	27.33
20	57.34	27.82	48.89	27.24
50	57.27	27.75	48.92	27.19
(b) Effect of Global Learning Rate				
1.00E-03	57.48	27.93	49.05	27.36
2.00E-03	57.4	27.76	48.83	27.18
3.00E-03	58.25	28.6	48.91	27.12
5.00E-03	58.37	28.69	49.98	28.13
0.01	57.33	27.56	48.74	27.07

Finally, we evaluated the sensitivity of FedGA to two key hyperparameters: the number of local epochs $K$ and the global learning rate $\eta _0$. As shown in Table 3, the model exhibits only minor fluctuations in performance across a wide range of settings. For example, although the best HR@20 and MRR@20 are observed when $K = 1$, increasing $K$ to 50 leads to less than a 2% drop in performance. Similarly, the optimal result occurs at $\eta _0 = 5 \times 10^{-3}$, but other values such as $1 \times 10^{-3}$ or $1 \times 10^{-2}$ still yield comparable results. These observations indicate that FedGA is robust to hyperparameter variations, retaining strong recommendation accuracy even when the settings are not precisely tuned. This robustness is especially valuable in real-world federated learning environments, where fine-grained tuning may be impractical.

In conclusion, the above results verify the performance of FedGA, and the effectiveness of the proposed strategies, especially for the non-IID data. In the next section, we explore the detailed contribution of each strategy and compare the proposed C-SME with the different SME strategies used in other adaptive FL baselines.

### Ablation Study (RQ2)

**Table 4:** Ablation study on Gowalla with SR-GNN.

Methods	HR@20	MRR@20	HR@10	MRR@10
FedGA - All	$58.57\pm 0.16$	$28.61\pm 0.07$	$50.14\pm 0.14$	$28.04\pm 0.08$
C-SME (w/o DRA)	$51.50\pm 0.12$	$24.21\pm 0.05$	$43.18\pm 0.13$	$23.60\pm 0.08$
FedYogi + DRA	$57.54\pm 0.05$	$28.01\pm 0.04$	$49.22\pm 0.03$	$27.43\pm 0.03$
FedAdam + DRA	$57.74\pm 0.02$	$28.03\pm 0.02$	$49.33\pm 0.04$	$27.44\pm 0.02$
FedYogi	$57.71\pm 0.12$	$27.94\pm 0.13$	$49.28\pm 0.12$	$27.35\pm 0.13$
FedAdam (S-SME)	$51.30\pm 0.18$	$24.02\pm 0.09$	$42.93\pm 0.15$	$23.44\pm 0.08$

To better understand the effectiveness and contribution of different strategies proposed in FedGA (RQ2), we performed an ablation study on C-SME and DRA in the Gowalla dataset and diagnosed these strategies. We compare the model performance of the full-version FedGA and three variants, namely C-SME (i.e., w/o DRA), FedYogi + DRA, and FedAdam + DRA. The results are reported in Table 4. It can be seen from the results that both DRA and C-SME have improved the performance of FL models differently. First, compared with FedAdam’s SME, C-SME has improved the model performance in most recommendation metrics and achieved better stability. Second, with the help of DRA, FedGA has achieved higher performance. It is worth noting that when DRA is used in FedYogi and FedAdam, the improvements are different. When DRA is used in FedAdam, the standard SME under DRA (i.e., FedAdam + DRA) has achieved a significant performance improvement (HR@20, MRR@20, HR@10, and MRR@10 have increased by 12.55%, 16.61%, 14.9%, and1 7.06%, respectively), and C-SME greatly improved the stability of the models. In FedYogi, DRA does not directly improve HR but slightly improves MRR, and dramatically improves the stability of GNN models. This is because FedYogi’s $\Delta \tilde{v}$ throughout the processes stays stable except for the point where $V=1$ (see Figure 3). Thus, the contribution of DRA is limited here. However, when $V=1$, with the help of DRA, the stability of FedYogi has been dramatically improved. Although C-SME outperforms FedAdam’s SME, it seems inferior to FedYogi’s SME when it is used alone. However, after combining C-SME with DRA, the overall performance is significantly better than that of FedYogi. Such an effect implies that these two strategies are not isolated, but instead they significantly promote and interact with each other. Combining all of these strategies always leads to better performance and stability comparable to that of FedYogi.

## Conclusion

This paper proposes a novel adaptive federated optimization method for session-based recommendation with GNN models. By incorporating Divergence-Resistant Aggregation (DRA) and Conditional Second-moment Estimation (C-SME), the proposed FedGA obtains efficient and stable performance over extreme non-IID data. Furthermore, we provided the theoretical convergence guarantees of FedGA and conducted empirical experiments on both open and industrial datasets to investigate FedGA’s performance. The experiment results demonstrate the effectiveness of our method. The motivation of proposing FedGA is to improve GNN’s efficiency for session recommendation. However, it is flexible to use FedGA for other neural networks and other FL use cases. Moreover, the proposed strategies can be used in conventional local adaptive optimizers, e.g., Adam, by simply modifying first- and second-moment estimations. Future directions include 1) exploring the dynamic methods for the second-moment estimation in adaptive federated optimization instead of manually chosen conditions based on the observations in FedGA, and 2) exploring how end-to-end FGL approaches can be adapted to SR scenarios. We also intend to contribute benchmark datasets specific to FL+SR scenarios (e.g., OpenFGL^46^) to help foster further research in federated GNN-SR modeling.

## Data Availability

Datasets used in experiments can be downloaded from: LastFM-1K: http://ocelma.net/MusicRecommendationDataset/lastfm-1K.html, Yoochoose: http://2015.recsyschallenge.com/challege.html, Retailrocket: https://www.kaggle.com/retailrocket/ecommerce-dataset, Gowalla: https://snap.stanford.edu/data/loc-Gowalla.html.
